# Altered Signaling in the G1 Phase Deregulates Chondrocyte Growth in a Mouse Model With Proteoglycan Undersulfation

**DOI:** 10.1002/jcb.24844

**Published:** 2014-05-13

**Authors:** Fabio De Leonardis, Luca Monti, Benedetta Gualeni, Ruggero Tenni, Antonella Forlino, Antonio Rossi

**Affiliations:** 1 Department of Molecular Medicine, Unit of Biochemistry, University of PaviaPavia, Italy

**Keywords:** GROWTH PLATE, CELL CYCLE, SKELETAL DYSPLASIA, PROTEOGLYCAN SULFATION, WNT PATHWAY

## Abstract

In several skeletal dysplasias defects in extracellular matrix molecules affect not only the structural and mechanical properties of cartilage, but also the complex network of signaling pathways involved in cell proliferation and differentiation. Sulfated proteoglycans, besides playing an important structural role in cartilage, are crucial in modulating the transport, diffusion, and interactions of growth factors with their specific targets, taking part in the regulation of signaling pathways involved in skeletal development and growth. In this work, we investigated by real time PCR and Western blots of the microdissected growth plate and by immunohistochemistry the molecular basis of reduced chondrocyte proliferation in the growth plate of the *dtd* mouse, a chondrodysplastic model with defective chondroitin sulfate proteoglycan sulfation of articular and growth plate cartilage. We detected activation of the Wnt pathway, leading to an increase in the non-phosphorylated form of nuclear β-catenin and subsequent up-regulation of cyclin D1 expression in the G1 phase of the cell cycle. β-Catenin was further stabilized by up-regulation of Smad3 expression through TGF-β pathway synergistic activation. We demonstrate that notwithstanding cyclin D1 expression increase, cell cycle progression is compromised in the G1 phase due to reduced phosphorylation of the pocket protein p130 leading to inhibition of transcription factors of the E2F family which are crucial for cell cycle progression and DNA replication. These data, together with altered Indian hedgehox signaling detected previously, explain at the molecular level the reduced chondrocyte proliferation rate of the *dtd* growth plate leading to reduced skeletal growth. J. Cell. Biochem. 115: 1779–1786, 2014.

The formation and growth of long bones and vertebral bodies are achieved through endochondral ossification, a strictly regulated process that requires a cartilage template from which bone develops in a coordinated manner. Essential to this process is the formation of the growth plate, a highly specialized cartilaginous structure responsible for bone elongation through rapid proliferation and differentiation of the chondrocytes forming it [Burdan et al., 2009].

The rate of proliferation and the maturation of proliferating into hypertrophic chondrocytes as well as the synthesis of regionally specific extracellular matrix (ECM) components is a multi-step, precisely timed, and spatially organized process regulated by several genetic, endocrine, and mechanical factors [Karsenty and Wagner, 2002; van der Eerden et al., 2003]. The ECM is composed of several types of collagens and proteoglycans, hyaluronic acid, link proteins, and other macromolecules, forming a highly organized network [Schwartz and Domowicz, 2002].

Proteoglycans play a key role in the development, morphology and function of the growth plate, as demonstrated by the several chondrodysplastic phenotypes in which a defect in core proteins or in the metabolism of chondroitin sulfate proteoglycans has been demonstrated. Mutations in aggrecan, the major cartilage proteoglycan, cause spondyloephimethaphyseal dysplasia or spondyloephiphyseal dysplasia; defects in the enzymes involved in proteoglycan synthesis result in Desbuquois dysplasia or Schenckenbecken dysplasia. Finally defects in sulfation result in spondyloephimethaphyseal dysplasia, PAPSS2 type, recessive Larsen syndrome, a musculo-skeletal variant of Ehlers-Danlos syndrome, and in the diastrophic dysplasia sulfate transporter (DTDST) family of disorders [Warman et al., 2010].

The DTDST disorders include diastrophic dysplasia, atelosteogenesis 2, achondrogenesis 1B, and recessive multiple epiphyseal dysplasia. These diseases are caused by mutations in the SLC26A2 (Solute Carrier family 26 member 2) whose functional impairment results in reduced sulfate uptake. Thus, the main biochemical effect of low intracellular sulfate levels is cartilage proteoglycan undersulfation. Previous studies demonstrated that articular cartilage from DTDST patients bears undersulfated glycosaminoglycans [Rossi et al., 1998], as well as the articular cartilage of *dtd* mice, a mouse model of diastrophic dysplasia [Forlino et al., 2005]. To study the cause of reduced long bone growth, we previously analyzed the growth plate of *dtd* mice, detecting consistent chondroitin sulfate proteoglycan undersulfation in all the different zones forming the growth plate compared to wild-type littermates [Gualeni et al., 2010; Mertz et al., 2012]. Interestingly, growth plate undersulfation affects the chondrocyte proliferation rate suggesting that the sulfation defect alters not only the ECM structure, but also the distribution of growth factors and signaling molecules throughout the growth plate. Since Indian hedgehog (Ihh), an important long range morphogen for chondrocyte proliferation, binds chondroitin sulfate proteoglycans [Cortes et al., 2009], we previously studied the distribution and the expression of Ihh in the growth plate of *dtd* mice, demonstrating that the sulfation defect does not affect Ihh expression, but its distribution in the growth plate. The altered distribution of this morphogen accounts, at least in part, for reduced skeletal growth in mutant animals [Gualeni et al., 2010].

However, a complex network of regulatory pathways has been described to act along with the parathyroid hormone-related peptide/Indian hedgehog (PTHrP/Ihh) negative feedback loop to control cartilage maturation, including fibroblast growth factor (FGF), bone morphogenetic protein (BMP), transforming growth factor-β (TGFβ), and Wnt signaling pathways [Ornitz and Marie, 2002; Yoon and Lyons, 2004; Kronenberg, 2006; Chun et al., 2008; Brochhausen et al., 2009]. The proper functioning of all these pathways relies on constitutive and extensive cross-interactions and influences chondrocyte proliferation and differentiation. The nature of such cross-talk among signaling pathways is overwhelmingly complex and context dependent. Sulfated proteoglycans, besides being important for the maintenance of the cartilage ECM mechanical properties, are also crucial in modulating the correct interactions between growth factors and their specific targets, allowing the regulation of signaling pathways involved in skeletal development in a time- and site-specific manner. All these pathways target specific cell cycle proteins, making the cell cycle machinery an integrator of the many extracellular signals regulating chondrocyte function [Beier, 2005].

On the basis of these observations, the reduced chondrocyte proliferation in the *dtd* growth plate might not be caused only by the PTHrP/Ihh pathway we described previously, but also by other signaling pathways. Thus, in this work we further investigate by real time RT-PCR, Western blot and immunohistochemistry the molecular basis of reduced chondrocyte proliferation in the *dtd* mouse growth plate. We demonstrate that, notwithstanding cyclin D1 expression increase, cell cycle progression was compromised in the G1 phase due to reduced phosphorylation of the pocket proteins leading to inhibition of the transcription factors of the E2F family.

## MATERIALS AND METHODS

### ANIMALS

The *dtd* mouse is a “knock-in” for a c1184t transition causing an A386V substitution in the eighth transmembrane domain of the Slc26a2, strongly reducing the activity of the transporter. In this study, wild-type and homozygous mutant mice with a C57Bl/6J × 129/SV background at postnatal day (P) 21 were used. Genotyping to distinguish mutant animals from heterozygous and wild-type littermates was performed either by PCR or Southern blotting, using genomic DNA extracted from mouse tail clips as previously described [Forlino et al., 2005].

Animals were bred with free access to water and standard pelleted food. Care and use of mice for this study were in compliance with animal welfare guidelines approved by the Animal Care and Use Committee of the University of Pavia.

### TISSUE PREPARATION

Tibiae from wild-type and mutant mice at P21 were excised and cleaned from the surrounding soft tissue. For real-time RT-PCR and protein analysis, tibiae were immediately included in optimal cutting temperature (OCT, Tissue-Tek) in RNase free conditions, frozen in dry ice, and stored at −80°C.

### GROWTH PLATE RNA EXTRACTION AND REAL TIME RT-PCR

Sixteen micrometer thick sections parallel to the long axis of the tibia were cut in RNase free conditions using a CM1850 cryostat (Leica) at −30°C and mounted on Superfrost Plus slides (Menzel-Glaser). Sections were fixed with decreasing concentration of ethanol, to remove OCT, and dehydrated with increasing concentration of ethanol and then washed in xylene. The growth plates were manually microdissected on the basis of cell morphology under a stereomicroscope (SMZ-143 Series, Motic) using an ophthalmic scalpel. Total RNA was immediately extracted from microdissected tissue using the RNAqueous-Micro Kit (Ambion), including the DNase digestion step according to the manufacturer's instructions. Quantitation and integrity of total RNA were determined by the Agilent Bioanalyzer 2100 (Agilent) with the Agilent RNA 6000 Pico Kit (Agilent). RNA (100 ng) was reverse transcribed using the High Capacity cDNA Archive Kit (Applied Biosystems) as per manufacturer's recommendation. For expression analyses by real time RT-PCR, the QuantiTect Primer Assays (QIAGEN) were used with the QuantiTect SYBR Green PCR Kit (QIAGEN) according to the manufacturer's protocol. Each sample was run in triplicate in 96 well plates in three independent experiments with the MX3000P (Stratagene) apparatus.

### WESTERN BLOT ANALYSIS

The microdissected growth plate obtained as described above was homogenized in 50 µl of Laemmli sample buffer (62.5 mM Tris–HCl, pH 6.8, 2% SDS, 10% (v/v) glycerol, 0.01% bromophenol blue) and the protein content was determined with the BCA Protein Assay (Pierce). Samples were then reduced in 5% β-mercaptoethanol, separated by 7% SDS–PAGE and blotted on a polyvinylidenedifluoride membrane (PVDF, Hybond). Primary antibodies used in this study were: anti-β-catenin, anti-cyclin D1, and anti-p107 (sc-7199, sc-753, and sc-318 respectively, Santa Cruz Biotechnology, Inc.), anti-p130 (610261, BD Transduction Laboratories™), anti-phospho-β-catenin (Ser33/37/Thr41, 9561, Cell Signaling Technology), and anti-β-actin (A5316, Sigma–Aldrich). The following secondary HRP-conjugated antibodies were used: donkey anti-rabbit IgG-HRP (sc-2077, Santa Cruz Biotechnology, Inc.) and sheep anti-mouse IgG-HRP (NA931, GE Healthcare). Four percent milk powder in 1× TBS-T (20 mM Tris–HCl, pH 7.5, 0.5 mM NaCl, and 0.1% (v/v) Tween-20) was used for blocking and for diluting antibodies. Chemiluminescence detection was achieved with the ECL Plus Western blotting detection reagents (GE Healthcare) and autoradiography films (Hyperfilm, GE Healthcare). Films were digitalized by VersaDoc™ Imaging System (Bio-Rad) and densitometric analysis was performed by Quantity One software (Bio-Rad).

### CONFOCAL ANALYSIS

Seven micrometer thick sections were cut parallel to the long axis of the tibia using a CM1850 cryostat at −30°C (Leica) and mounted on Superfrost Plus slides (Menzel-Glaser). Sections were hydrated with decreasing concentration of ethanol, to remove OCT, fixed in neutral buffered formalin solution and washed with 0.5 M glycine in PBS. Sections were digested with 1 mg/ml hyaluronidase from sheep testes (Sigma–Aldrich) in 0.15 M sodium chloride, 0.1 M sodium acetate, pH 5.5, for 45 min at 37°C. 5% BSA, 0.05% Tween-20 (v/v) in PBS was used for blocking, and 0.05% Tween-20 (v/v) in PBS for washing steps. The following primary antibodies diluted 1:100 in 1% BSA, 0.05% Tween-20 in PBS were used: anti-β-catenin (sc-7199, Santa Cruz Biotechnology, Inc.) and anti-phospho-β-catenin (Ser33/37/Thr41, 9561, Cell Signaling Technology). Secondary antibody was Alexa Fluor® 647 goat anti-rabbit (Life Technologies). The nuclei were counterstained by 0.5 μg/ml 4,6-diamidino-2-phenylindole (DAPI; Sigma–Aldrich). Slides were mounted using Mowiol (Sigma–Aldrich). The sections were examined by TCS SP2-Leica confocal microscope (Leica). Co-localization was evaluated on single planes.

### STATISTICAL ANALYSIS

All results of real time RT-PCR and densitometric analysis of Western blots were compared between the *dtd* and wild-type littermates with the Student's *t*-test using SigmaPlot® 11 software; *P* values ≤0.05 were considered significant.

## RESULTS

### CYCLIN D1 IS OVEREXPRESSED IN THE GROWTH PLATE OF DTD MICE

Previous studies on the tibial growth plate demonstrated reduced chondrocyte proliferation rate in the *dtd* growth plate at P21 compared to age matched controls [Gualeni et al., 2010]. In order to study its molecular basis, we considered the expression of the main players involved in cell cycle progression. Expression levels were determined by real time RT-PCR on total RNA isolated from the microdissected growth plate.

On the first instance, we measured the expression levels of the main cyclins involved in the progression of each phase of the cell cycle (Table1). The cyclins E, A, and B (controlling the progression through the G1/S, S/G2, and mitosis phases, respectively) did not show statistically significant expression differences between *dtd* and wild-type mice. Among the three isoforms of cyclin D involved in the G1 phase, cyclin D1 showed a significant increase in *dtd* mice by approximately 2.5-fold compared to wild-type mice (*P* < 0.05). Also cyclin dependent kinases (CDKs) 4 and 6 that interact with cyclin D1 showed an higher expression in the *dtd* versus wild-type littermates: Cdk6 expression was increased in *dtd* mice by approximately 2.5-fold compared to wild-type mice (*P* < 0.05), while Cdk4 had the same trend even if the difference was not statistically significant.

**I:** Gene Expression Analysis

Gene	Description	dtd/wt
Cell cycle		
Smad1	SMAD family member 1	1.87
Smad2	SMAD family member 2	1.19
Smad3	SMAD family member 3	2.50
Ccnd1	Cyclin D1	2.42
Ccnd2	Cyclin D2	1.31
Ccnd3	Cyclin D3	0.93
Ccne1	Cyclin E1	0.76
Ccna2	Cyclin A2	0.67
Ccnb1	Cyclin B1	0.72
Cdk4	Cyclin-dependent kinase 4	1.55
Cdk6	Cyclin-dependent kinase 6	2.40
Cdk7	Cyclin-dependent kinase 7	1.26
Cdc25a	Cell division cycle 25 homolog A	1.07
E2f1	E2F transcription factor 1	0.73
E2f2	E2F transcription factor 2	0.79
E2f3	E2F transcription factor 3	1.47
E2f4	E2F transcription factor 4	1.09
E2f5	E2F transcription factor 5	1.45
Wnt signaling pathway		
Wnt4	Wingless- type MMTV integration site family, member 4	0.94
Wnt5a	Wingless- type MMTV integration site family, member 5a	1.34
Wnt5b	Wingless- type MMTV integration site family, member 5b	0.93
Wnt9a	Wingless- type MMTV integration site family, member 9a	1.73
Wisp1	Wnt1 inducible signaling pathway protein 1	2.29
Wisp2	Wnt1 inducible signaling pathway protein 2	1.03
Wif1	Wnt inhibitory factor 1	2.85
Sfrp1	Secreted Frizzled Related Protein 1	1.87
Sfrp2	Secreted Frizzled Related Protein 2	1.22
Sfrp3	Secreted Frizzled Related Protein 3	1.37
Sfrp4	Secreted Frizzled Related Protein 4	0.30
Dkk	Dickkopf homolog 1	2.39
Fzd1	Frizzled homolog 1	1.48
Fzd2	Frizzled homolog 2	1.13
Fzd5	Frizzled homolog 5	5.85
Fzd7	Frizzled homolog 7	1.62
Lrp5	Low density lipoprotein receptor-related protein 5	2.39
Lrp6	Low density lipoprotein receptor-related protein 6	1.37
Ctnnb1	β-Catenin	2.18
Tcf7	Transcription factor 7, T-cell specific	2.50
Lef1	Lymphoid enhancer binding factor 1	1.02
Transcription factors of chondrocyte and osteoblast differentiation		
Runx2	Runt related transcription factor 2	1.64
Sox2	SRY-box containing gene 2	1.77
Sox9	SRY-box containing gene 9	0.95

Real time RT-PCR experiments on total RNA isolated from the microdissected growth plate were performed on samples from wild-type (wt) and mutant (*dtd*) mice at P21. Three *dtd* and three wild-type mice were used. Each sample was run in triplicate and three different experiments were performed. Genes are classified according to their biological role. Numbers represent fold induction in *dtd* relative to wild-type expression for each gene. A twofold expression difference was considered significant when *P* < 0.05. Significant induction of expression in *dtd* mice versus wt is shown as boldface; no significant down-regulation was observed in this gene panel.

**Figure 1:**
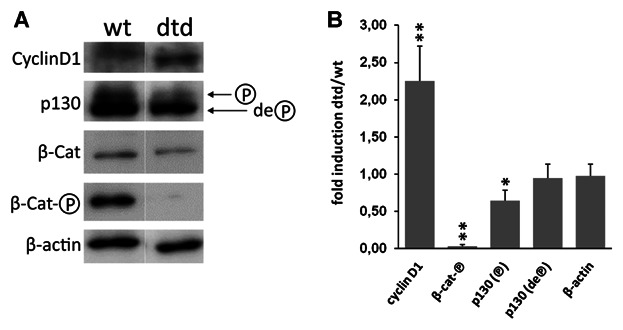
Western blot and densitometric analysis of relevant proteins involved in the regulation of the G1 phase of the cell cycle in the growth plate of wild-type (wt) and mutant (*dtd*) P21 mice. A: Blotting membranes were incubated with antibodies raised against cyclin D1, the phosphorylated and de-phosphorylated form of p130, β-catenin and the phosphorylated form of β-catenin (β-Cat and β-Cat-℗, respectively). B: The expression of each protein was quantitated by densitometric analysis of Western blots and normalized to β-actin. Numbers represent fold induction in *dtd* relative to wild-type expression of each protein. The microdissected growth plate of three *dtd* and three wild-type mice were used and each sample was run in triplicate; mean values ± SD are reported in the graph. *P < 0.05; **P < 0.001.

The up-regulation of cyclin D1 gene in mutant mice was confirmed at the protein level by Western blot analysis on protein extracts of the microdissected growth plate: densitometric analysis reported a twofold increase in *dtd* compared to wild-type littermates (Fig. 1).

### WNT AND TGF-β PATHWAYS ARE ACTIVATED IN DTD MICE

Cyclin D1 expression is regulated by a large number of extracellular growth factors and hormones with known mitogenic roles in chondrocyte proliferation including PTHrP/Ihh, TGF-β, and Wnt [Beier, 2005]. Since we have already analyzed the PTHrP/Ihh signaling pathway in a previous study [Gualeni et al., 2010], here we considered the activation of the Wnt canonical pathway, focusing in particular on the effectors that are known to be involved in skeletal disorders or in chondrocyte anomalies [Chun et al., 2008] (Table1). The ligands did not show statistically significant differences between *dtd* and wild-type animals while, among the antagonists, Wisp1, Wif1, and Dkk1 exhibited a statistically significant increase in *dtd* mice compared to wild-type littermates as well as the receptor Fzd5 and the co-receptor Lrp5. In particular, Fzd5 showed a fivefold increase in mutant animals compared to wild types. The expression of the key element of the Wnt canonical pathway, β-catenin, was increased more than twofold in *dtd* mice compared to wild-types as well as the transcription factor Tcf7, the target of this specific pathway. Among the genes targeted in the canonical pathway there are regulators of cell cycle progression, in particular cyclin D1 as observed above [Yang et al., 2003].

Similarly the protein level of β-catenin as assessed by Western blotting was significantly higher in wild-type mice compared to mutants. The higher relative levels of the phosphorylated form of β-catenin indicate more extensive degradation in wild-type animals compared to *dtd* littermates (Fig. 1).

In the Wnt/β-catenin canonical pathway, the binding of a Wnt family ligand causes the destabilization of the “destruction complex” [MacDonald et al., 2009]. This event prevents β-catenin phosphorylation causing the reaching of an intracellular concentration threshold that allows its translocation to the nucleus where it acts as a co-activator for TCF. The ratio phospho-β-catenin/β-catenin was 1.118 ± 0.189 and 0.083 ± 0.061 (mean ± SD) in wild-type and *dtd*, respectively (*P* < 0.001) suggesting the activation of Wnt signaling in mutant mice.

**Figure 2:**
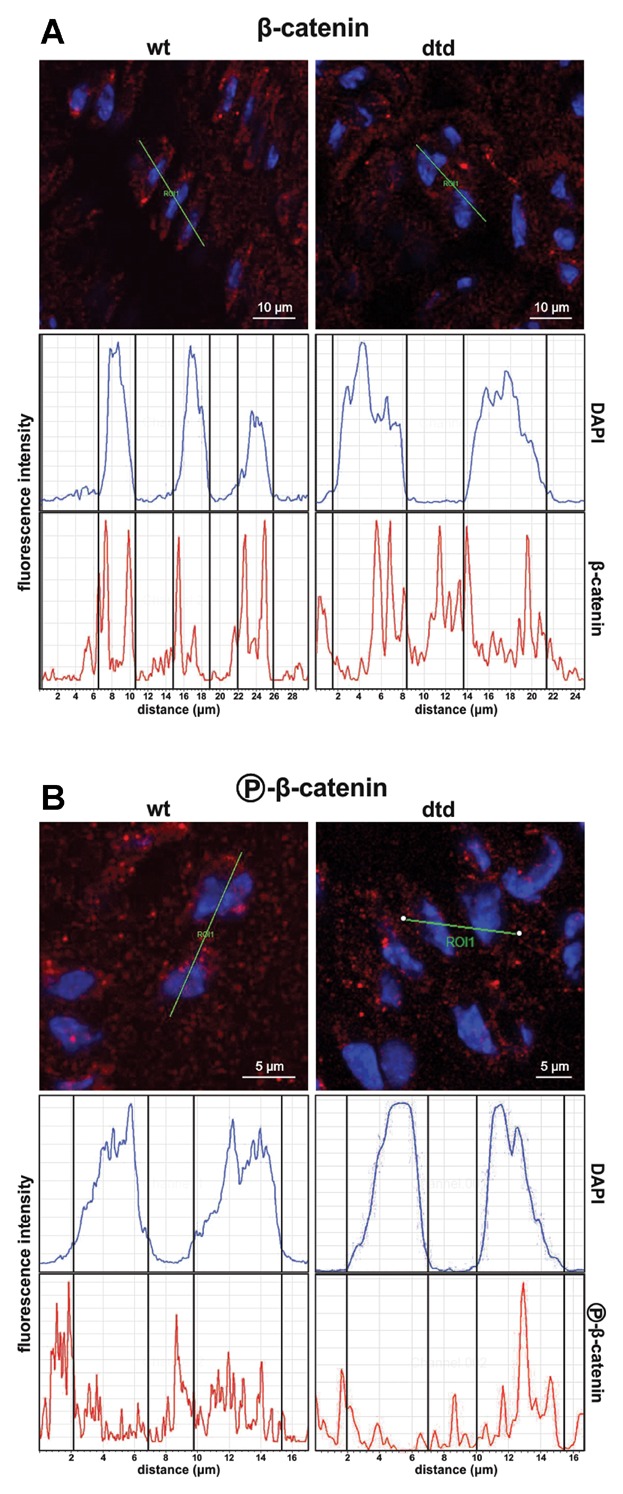
Confocal microscopy of intracellular β-catenin distribution in chondrocytes of the proliferative zone. Cryosections of the tibial growth plate were incubated with anti-β-catenin (A) or anti-phospho-β-catenin (B) antibodies followed by Alexa Fluor® 647 conjugated secondary antibody. Nuclei were stained with DAPI. The green bars represent the selected points in the proliferative zone for semiquantitative immunohistochemical analysis of the two fluorescence labeling shown in the graphs. Total β-catenin was localized in the cytosol and in the nucleus of chondrocytes, while the phosphorylated β-catenin (undergoing degradation) was present only in the cytosol of wild-type cells. Five *dtd* and five wild-type mice were used; an average of 6 sections per animal was considered and 20 proliferative columns per section were analyzed.

To further confirm the activation status of this signaling pathway, we looked for the intracellular distribution (cytosol vs. nucleus) of β-catenin in growth plate chondrocytes of *dtd* and wild-type littermates by confocal microscopy; we also considered the localization of the phospho-β-catenin, using a specific antibody against the phosphorylated form. As reported in Figure 2A, total β-catenin was localized in the cytosol and in the nucleus of chondrocytes, while the phosphorylated β-catenin (undergoing degradation) was present only in the cytosol of wild-type cells (Fig. 2B). This result confirms the activation of the canonical pathway, since the β-catenin was stabilized and translocated to the nucleus of *dtd* chondrocytes. β-Catenin activation and its nuclear translocation was further supported by Smad3 analysis; in fact, Smad3 expression was increased by 2.5-fold in *dtd* chondrocytes compared to wild-type cells. Increased Smad3 expression suggested activation of the TGFβ pathway.

### GROWTH INHIBITION OF DTD CHONDROCYTES IN THE G1 PHASE IS MEDIATED BY POCKET PROTEIN DEPHOSPHORYLATION

The activity of the E2f family of transcription factors (E2fs) is negatively controlled by physical association of the dephosphorylated pocket proteins that are the pRb and two related proteins, p107 and p130. In particular, it has been demonstrated in vitro that p107 dephosphorylation is involved in the early stage of chondrocyte growth arrest, while p130 is involved in the maintenance of growth arrest at the steady state [Dailey et al., 2003]. Thus, since in our in vivo model growth alteration can be considered at the steady state we focused on p130 and we assessed its expression level by Western blotting. We concentrated on protein phosphorylation, since the mechanism of action of p130 is related to the phosphorylation level, rather than to its whole expression. Phosphorylation of p130 was significantly reduced in *dtd* mice compared to wild-types, while the level of dephosphorylated p130 was normal, suggesting a disequilibrium in favor of the inhibition state (Fig. 1). This finding was confirmed by the expression analysis of E2f target genes such as cyclin E, cyclin A, Cdc25a that were not up-regulated in *dtd* animals (Table1). Finally, the levels of E2fs (in particular E2f1 and E2f4) which are expressed in chondrocytes [Ito et al., 2014] and associate with pocket proteins were not noticeably different between wild-type and *dtd* mice (Table1). Together, these observations suggest that in spite of cyclin D1 and Cdk6 overexpression in mutant mice, the expression of genes required for DNA replication were not similarly up-regulated, suggesting that *dtd* chondrocytes undergo cell cycle down-regulation during the entry in the S-phase.

## DISCUSSION

Previous studies targeted at elucidating the molecular basis of reduced long bone growth in *dtd* mice demonstrated that chondroitin sulfate proteoglycan undersulfation in the growth plate zones (resting, proliferative, and hypertrophic zones) and reduced chondrocyte proliferation rate in the proliferative zone of mutant mice compared to wild-types account for a reduced number of cells per column in the proliferative and consequently in the hypertrophic zone of *dtd* animals, resulting in overall reduction of long bone growth [Gualeni et al., 2010; Mertz et al., 2012]. Reduced cell proliferation was linked to an altered distribution of Ihh in the growth plate in mutant mice since its diffusion and signaling is mediated by sulfation of chondroitin sulfate proteoglycans [Cortes et al., 2009; Gualeni et al., 2010]. However, it is well known that other signaling pathways, such as FGF, TGF-β, Wnt, and BMP, play an active role in regulating chondrocyte proliferation and differentiation [Beier, 2005]. To identify which pathways beside Ihh could account for reduced chondrocyte proliferation in the growth plate of *dtd* mice we analyzed the expression of cell cycle genes, since they play a crucial role in the integration and translation of various extracellular signals.

Progression through the cell cycle is controlled by sequential activation of several cyclin-dependent kinases after association with specific cyclins. Cyclin E, A, and B expressions did not show significant differences in mutant compared to wild-type mice even if a trend toward lower expression was observed in *dtd* animals. Conversely, cyclin D was upregulated both at the transcription and protein level, suggesting increased proliferation, in contrast with reduced chondrocyte proliferation detected in vivo in the growth plate of P21 *dtd* mice.

**Figure 3:**
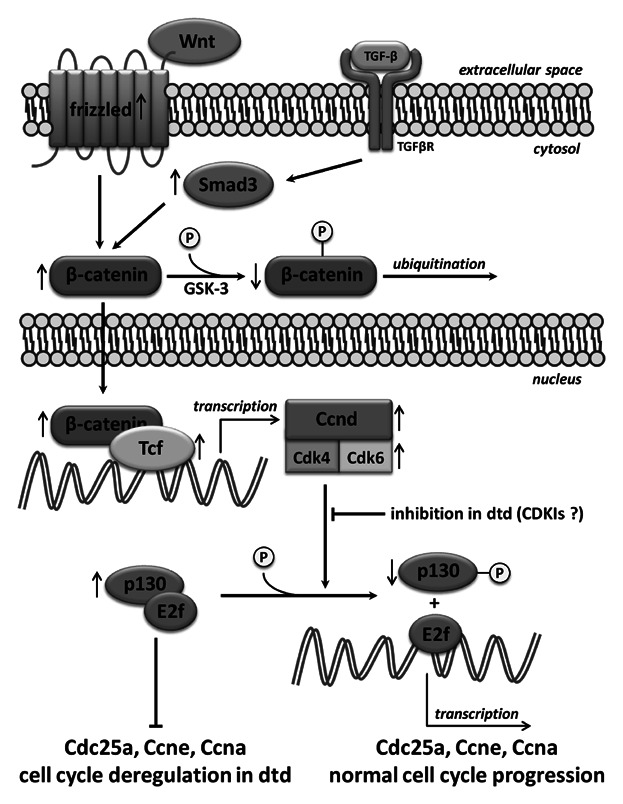
Schematic presentation of mechanisms leading to cell cycle deregulation in growth plate *dtd* chondrocytes. Wnt canonical pathway activation and TGF-β activation through Smad3 lead to the stabilization of β-catenin resulting in cyclin D (Ccnd) and Cdk6 increase expression pointing to an activation of cell cycle progression in *dtd* mice. However the Ccnd–Cdk complex, in spite of its up-regulation, is not able to phosphorylate p130; weak p130 phosphorylation prevents E2f release from the pocket protein. This step, which is crucial to activate transcription of target genes involved in cell cycle progression (Cdc25a, Ccne, and Ccna), explains cell cycle deregulation in the G1 phase of *dtd* chondrocytes. Arrows pointing up or down indicate gene/protein up- or down-regulation in *dtd* mice, respectively.

Since withdrawal from the cell cycle during differentiation occurs in G1, as does the decision to continue proliferation, we focused our studies on the G1 phase of the cell cycle and we considered the effectors upstream and downstream cyclin D. In fact D type cyclins, in particular the D1 isoform which is growth plate specific [Long et al., 2001; Yang et al., 2003], are absolutely required for passage through G1 and they might act as integrators of multiple extracellular stimuli and as transducers of these signals to the cell cycle machinery [Fu et al., 2004; Beier, 2005]. A model depicting the molecular basis of deregulated cell cycle progression in the G1 phase of *dtd* chondrocytes is shown in Figure 3.

Wnt and TGF-β are well-known extracellular growth factors; the cross-talk between them occurs in the nucleus where the Smad/β-catenin/TCF protein complex regulates and promotes gene transcription (including cyclin D) [Guo and Wang, 2009]. In fact Wnt activation prevents β-catenin phosphorylation and thus proteasomal degradation, and favors its translocation to the nucleus to activate TCF. TGF-β acts in a synergistic way by increasing Smad3 expression which prevents β-catenin degradation and facilitates its nuclear translocation [Li et al., 2006; Zhang et al., 2010]. For this reason we considered β-catenin expression levels and, most crucial, the ratio between the phosphorylated form, which is targeted to degradation by the proteasome, and the non-phosphorylated form that activates Tcf7. In *dtd* mice, a higher relative amount of the non-phosphorylated form was detected compared to wild type mice, in agreement with increased expression of Tcf7 and cyclin D. At the mRNA level, we detected increased expression of β-catenin in *dtd* mice, but this finding did not parallel the protein results from densitometric analysis of Western blots. There are evidence that β-catenin expression is post-transcriptionally regulated by proteins that stabilize the mRNA such as the Zipcode Binding Protein 1 [Gu et al., 2008]; these might explain the discrepancy observed in β-catenin expression at the mRNA and protein level. Few data are available regarding β-catenin expression at the transcriptional level, while a huge bulk of evidence have pointed out that signaling through β-catenin is regulated by modulating its degradation and nuclear translocation rather than its absolute amounts; moreover, it has been reported that fold change (post-Wnt/pre-Wnt stimulation) and not absolute level of β-catenin dictates Wnt signaling [Goentoro and Kirschner, 2009]. In conclusion, expression studies of the Wnt pathway suggest activation of the Wnt canonical pathway in *dtd* mice; this observation, despite the up-regulation of some genes coding for inhibitors of the Wnt/β-catenin canonical pathway, is supported by the up-regulation of the receptor and the co-receptor Fzd5 and Lrp5, respectively, and of Tcf7 mRNA and by the increased relative amount of the non-phosphorylated form of β-catenin localized in the nucleus.

Finally, increased expression of Smad3 suggests activation of the TGF-β pathway. All these data point to an activation of the cell cycle in *dtd* mice, but are in contrast with the reduced chondrocyte proliferation rate observed in the *dtd* growth plate [Gualeni et al., 2010] and with the normal transcription level of cyclins E, A, and B involved in the other phases of the cell cycle.

Beside the control of the cell cycle by successive activation of several CDKs, a second level of regulation is provided by phosphorylation of the pocket proteins (pRb Retinoblastoma protein, p107 and p130). In fact hyperphosphorylation of pocket proteins releases E2F from the pocket protein and induces expression of E2F target genes necessary for cell cycle progression, DNA replication and mitotic activity, such as cyclin A, cyclin E, and Cdc25a [Vigo et al., 1999; Wells et al., 2000]. Thus, pocket proteins play an essential role in cell cycle control as mediators between the extracellular signal-regulated cyclin-CDK activity and the transcription factors controlling expression of genes necessary for DNA replication and cell cycle progression. Among pocket proteins p107 and in particular p130 seem to have important functions in chondrocytes as demonstrated by mice lacking functional p107 or p130 [Cobrinik et al., 1996]; p107 dephosphorylation is very fast and it is independent from changes in the composition of the cyclin–Cdk complexes suggesting that this protein is involved in the initiation of the growth arrest; p130 is involved in the maintenance of the growth arrest and is also required to elicit maximal level of growth arrest [Dailey et al., 2003]. In *dtd* mice, the phosphorylated form of p130 was significantly reduced compared to wild-type mice indicating that the unphosphorylated species can form complexes with transcription factors of the E2f family. Consistent with this finding the expression of E2f dependent proteins cyclin A and cyclin E effectors of the G1-S transition [Cobrinik, 2005] as well as Cdc25a, a tyrosine phosphatase essential for the entry in the S phase [Vigo et al., 1999], was not up-regulated in *dtd* mice, suggesting that chondrocytes failed to accomplish the cell cycle due to deregulation of E2f target genes in spite the up-regulation of cyclin D1 and Cdk6. The same findings have been reported recently by Ito et al in a *Ccnd1*/*Cdk6* double transgenic mouse model in which cyclin D1 and Cdk6 were overexpressed in cartilage, but E2f target genes were significantly down-regulated because of the up-regulation of dephosphorylated p107. As a result mice showed dwarfism with shortened limbs, domed skull, small thoracic cage, and thin ribs and vertebrae [Ito et al., 2014].

Cell cycle arrest despite the increase of cyclin D1 expression have been reported in primary chondrocyte cultures from patients with thanatophoric dysplasia type I, a lethal chondrodysplasia caused by activating mutations of the FGFR3 [Laplantine et al., 2002; Dailey et al., 2003; Schibler et al., 2009]. Furthermore Dailey et al. by studying the molecular basis of the inhibitory effect of FGF on chondrocyte proliferation using rat chondrosarcoma chondrocytes demonstrated that FGF induced a rapid dephosphorylation of pocket proteins and arrest of the cells in the G1 phase of the cell cycle. By incubating cells with FGF at different time points, they demonstrated that among pocket proteins p107 and p130 but not pRb, are critical effectors of FGF-mediated growth inhibition in chondrocytes; in particular p107 is mainly involved in the initiation of growth arrest, while p130 dephosphorylation in the maintenance of growth arrest [Laplantine et al., 2002; Dailey et al., 2003; Schibler et al., 2009].

Pocket proteins bind to transcription factors of the E2f family, and their phosphorylation by cyclin D1 and Cdks results in E2f dissociation. However, p107 and p130 preferentially bind to different E2fs from those bound by pRb, and are required for the regulation of distinct E2f-responsive genes [Cobrinik, 2005]. Thus the unbalanced regulation of cell cycle activators (cyclin D1/Cdk complex), the altered ratio of phosphorylated vs. dephosphorylated form of pocket proteins might account for the failure in a corresponding up-regulation of E2f target genes in *dtd* mice chondrocytes.

In conclusion, we suggest that in our mouse model, in spite of the activation of the Wnt and TGF-β signaling pathway promoting cell proliferation, chondrocytes were blocked in the G1 phase and could not proceed through the S phase because of E2F inability to induce transcription of genes necessary for cell cycle progression and DNA replication.
